# Blueberry Peel Extracts Inhibit Adipogenesis in 3T3-L1 Cells and Reduce High-Fat Diet-Induced Obesity

**DOI:** 10.1371/journal.pone.0069925

**Published:** 2013-07-25

**Authors:** Yuno Song, Hyoung Joon Park, Suk Nam Kang, Sun-Hee Jang, Soo-Jung Lee, Yeoung-Gyu Ko, Gon-Sup Kim, Jae-Hyeon Cho

**Affiliations:** 1 Institute of Life Science, College of Veterinary Medicine, Gyeongsang National University, Jinju, Korea; 2 Deptment of Foods and Nutrition, Gyeongsang National University, Jinju, Korea; 3 Dept. of Animal Science & Biotechnology, Gyeongnam National University of Science and Technology, Jinju, Korea; 4 Animal Genetic Resources Station, National Institute of Animal Science, RDA, Namwon, Korea; National Institute of Nutrition, India

## Abstract

This study examined the anti-obesity effect and mechanism of action of blueberry peel extracts (BPE) in 3T3-L1 cells and high-fat diet (HFD)-induced obese rats. The levels of lipid accumulation were measured, along with the changes in the expression of genes and proteins associated with adipocyte differentiation in 3T3-L1 cells. Evidenced by Oil-red O staining and triglyceride assay, BPE dose-dependently inhibited lipid accumulation at concentrations of 0, 50, and 200 µg/ml. BPE decreased the expression of the key adipocyte differentiation regulator C/EBPβ, as well as the C/EBPα and PPARγ genes, during the differentiation of preadipocytes into adipocytes. Moreover, BPE down-regulated adipocyte-specific genes such as aP2 and FAS compared with control adipocytes. The specific mechanism mediating the effects of BP revealed that insulin-stimulated phosphorylation of Akt was strongly decreased, and its downstream substrate, phospho-GSK3β, was downregulated by BPE treatment in 3T3-L1 cells. Together, these data indicated that BP exerted anti-adipogenic activity by inhibiting the expression of PPARγ and C/EBPβ and the Akt signaling pathway in 3T3-L1 adipocytes. Next, we investigated whether BP extracts attenuated HFD-induced obesity in rats. Oral administration of BPE reduced HFD-induced body weight gain significantly without affecting food intake. The epididymal or perirenal adipose tissue weights were lower in rats on an HFD plus BPE compared with the tissue weights of HFD-induced obese rats. Total cholesterol and triglyceride levels in the rats fed BPE were modestly reduced, and the HDL-cholesterol level was significantly increased in HFD plus BP-fed rats compared with those of HFD-fed rats. Taken together, these results demonstrated an inhibitory effect of BP on adipogenesis through the down-regulation of C/EBPβ, C/EBPα, and PPARγ and the reduction of the phospho-Akt adipogenic factor in 3T3-L1 cells. Moreover, BPE reduced body weight gain and inhibited fat accumulation in an HFD-induced animal model of obesity.

## Introduction

Obesity is one of the greatest public health problems and major risk factors for serious metabolic diseases and significantly increases the risk of premature death. Obesity arises from an imbalance in energy intake and energy expenditure that eventually leads to the pathological growth of adipocytes [1]. Adipocytes are the major cellular component of fatty tissue. Excess fat is accumulated in adipocytes as excessive amounts of lipids (triglycerides), resulting in elevated triglyceride content in plasma and tissues like liver and muscle, which leads to pathological dysfunction of these tissues [2], [3].

Fat accumulation and adipocyte differentiation are associated with the occurrence and development of obesity [4]. Adipocyte differentiation depends upon the coordinated regulation of gene expression. Adipogenic transcription factors such as of the CCAAT/enhancer binding protein-beta (C/EBPβ), nuclear receptor peroxisome proliferator-activated receptor gamma (PPARγ), and CCAAT/enhancer binding protein-alpha (C/EBPα) play a key role in the complex transcriptional cascade that occurs during adipogenesis [5]. C/EBPβ is induced immediately after differentiation, whereas C/EBPα and PPARγ are expressed much later [5], [6]. They are necessary for the expression of adipocyte-specific genes, such as adipocyte fatty acid-binding protein (aP2), lipoprotein lipase (LPL), leptin, adiponectin and fatty acid synthase (FAS) [5], [7], which lead to morphological changes and lipid accumulation within the cells. It is well established that the activation of the serine/threonine kinase Akt pathway plays a major role in adipocyte differentiation in which insulin and certain growth factors stimulate adipogenesis. Moreover, the overexpression of constitutively active Akt increases glucose uptake and adipocyte differentiation in 3T3-L1 adipocytes [8]. Akt phosphorylates and regulates a number of substrates involved in a diverse array of biological processes [9] and is essential to induce PPARγ expression [10]. Glycogen synthase kinase 3 beta (GSK3β) is a critical downstream signaling protein of the phosphoinositide 3-kinase (PI3K)/Akt pathway. Insulin signaling activates Akt through PI3K and induces serine/threonine phosphorylation of downstream target, GSK3β, which phosphorylates C/EBPβ, C/EBPα, and glycogen synthesis (GS) [11], [12].

Despite the short-term benefits of treating obesity with drugs, medication-induced weight loss is often associated with negative side effects and rebound weight gain when the medications are discontinued [13]. Thus, new research into healthy foods or drugs without negative side effects is required for the prevention and therapy for obesity. Recently, it was reported that natural compounds from plants, such as herbal medicines and their derivatives, can help treat obesity without noticeable adverse effects or mortality [14], [15]. Blueberries (BB) are one of the most popular fruits and are also rich in polyphenols [16]. BB polyphenols have shown promising results treating cognitive impairment, ischemic heart disease, oxidative stress, and neurological degeneration [17], [18]. Ethanol extracts from the BB leaf, stem, root, and fruits contained active compounds with insulin-like and glitazone-like properties and protected against glucose toxicity [19]. In obese people, the consumption of BB improved metabolism at dietary achievable doses [20]. BB consumption is associated with various health benefits. However, it remains unknown how Blueberry peel (BP) promote an anti-obesity effect in 3T3-L1 adipocytes and high fat diet (HFD)-induced obese rats.

In the present study, we examined the mechanism of Blueberry peel extract (BPE)-induced adipocyte differentiation and adipogenesis in 3T3-L1 cells. Moreover, we evaluated the influence of BPE on body weight, epididymal fat and perirenal fat weight, and lipid profiles in obese rats fed a high-fat diet.

## Materials and Methods

### Preparation of Blueberry Peel Extracts (BPE)

The blueberries were immediately peeled after harvested from 10 to 20 September 2011 at Sanchung, Gyeongnam (Animal Bio-Resources Bank, Gyeongnam, Korea). Blueberry peels (BP), a by-product from ready-to-eat vegetable and jam industry, were obtained from Dulleya Co., Ltd. (Gyeongnam, Korea) and kept at −18°C until use. For the sample preparation, the blueberry peels were air-dried at 50°C (air velocity 1.5 m/s) for 72 h and ground into a fine powder using a Waring blender (51 BL 32, Torrington, CT, USA) for 10 min at high speed and stored at −18°C before any further treatments. The powdered peel (500 g) of blueberries was soaked in 2500 ml water for 48 h and then refluxed for another 12 h at 80°C. The supernatant was collected and ethanol was added to 80% (vol/vol). The extracts were stored at room temperature for 48 h, filtered through chromatography paper (Whatman No 3, UK), and then 200 ml of supernatant was mixed with 800 ml 80% ethanol. Extracts were incubated at room temperature for 24 h and were centrifuged at 3000 rpm at 4°C for 10 min. The supernatant was evaporated using rotator evaporator (Heidolph, Schwabach, Germany) at 50°C. The extracts were diluted in H_2_O and stored at −20°C overnight and freeze-dried and powdered. For analysis of bioactive characteristics, blueberry peel extracts were dissolved in distilled water at a concentration of 500 mg/ml and stored at −20°C until further analysis.

### Measurement of Total Phenolic Content using Folin-Ciocalteu Assay

The total phenolic content of the BPE was determined using a spectrophotometer according to the Folin-Ciocalteu colorimetric method [21]. Because quercetin is one of the polyphenol compounds found in BB, the total phenolic content of ethanol extract of BB was expressed as mg quercetin (Sigma-aldrich, USA) equivalents (QE)/g.

### Measurement of Total Flavonoids

The total flavonoid content was determined as previously described [22] with slight modifications. Briefly, 0.25 mL of BPE (100 µg/mL) was added to a tube containing 1 mL of double-distilled water. Next, 0.075 mL of 5% NaNO_2_, 0.075 mL of 10% AlCl_3_ and 0.5 mL of 1 M NaOH were added sequentially at 0, 5 and 6 min. Finally, the volume at the reacting solution was adjusted to 2.5 mL with double-distilled water. The solution had an absorbance of 410 nm that was detected using an Ultrospec 2100 Pro Spectrophotometer (Section 3.3). The results were expressed in mg quercetin equivalents (QE)/g.

### Measurement of Free Radical Scavenging Activity on DPPH Assay

The free radical scavenging activity of BPE (100 µg/mL in DW) was measured using the method of Brand-Williams [23] with some modification. L-ascorbic acid was used as a positive control. The inhibition percentage was calculated from the following equation: Inhibition % = [(absorbance of control-absorbance of sample)/absorbance of control]×100. The absorbance was measured by a spectrophotometer (Ultrospec 2100 pro; Amersham Pharmacia Biotech Co., Piscataway, NJ, USA).

### Measurement of Superoxide Anion (O2^•−^) Radical Scavenging and Hydroxyl (OH^•^) Radical Scavenging Activity

Superoxide radicals were generated by a method described in a previous paper [24]. The samples (100 ug/mL in DMSO) were added to the reaction solution containing 100 µL of 30 mM EDTA (pH 7.4), 10 µL of 30 mM hypoxantine in 50 mM NaOH, and 200 µL of 1.42 mM nitroblue tetrazolium (NBT). After the solution was preincubated at room temperature for 3 min, 100 µL of 0.5 U/mL xanthine oxidase was added to the mixture and the volume was brought upto 3 ml with 50 mM phosphate buffer (pH 7.4). After the solution was incubated at room temperature for 20 min, absorbance was measured at 560 nm. The reaction mixture without xanthine oxidase was used as a blank (A1). The samples (A2) were added to the reaction mixture, in which O2^•−^ was scavenged, thereby inhibiting the NBT reduction. Absorbance was measured and the decrease in O2^•−^ was represented by A2-A1. Quercetin was used as a positive control. The scavenging activity on superoxide anion radical (SRSA) was calculated by the following equation: SRSA % = (A2 − A1/A1) ×100. The scavenging activity of samples (100 µg/mL) in DMSO on the hydroxyl radical (OH^•^) was measured by the deoxyribose method [25] with a slight modification. The deoxyribose assay was performed in 10 mM phosphate buffer (pH 7.4) containing 2.5 mM deoxyribose, 1.5 mM H_2_O_2_, 100 µM FeCl_3_, 104 µM EDTA, and the test sample (0.5 mg/mL). The reaction was started by adding ascorbic acid to a final concentration of 100 µM. The reaction mixture was incubated for 1 h at 37°C in a water-bath. After incubation, the color was developed by addition of 0.5% thiobarbituric acid followed by ice-cold 2.8% trichloroacetic acid in 25 mM NaOH and heating for 30 min at 80°C. A control was performed without samples (A1). The sample (A2) was cooled on ice and the absorbance was measured at 532 nm. The hydroxyl radical scavenging activity (HRSA) was calculated by the following equation: HRSA% = (A1− A2/A1) ×100.

### Cell Culture

Mouse 3T3-L1 preadipocytes were grown in Dulbecco’s modified eagle medium (DMEM) high glucose with 10% calf serum at 37°C in a humidified atmosphere of 5% CO_2_. At 1 day postconfluence (designated “day 0”), cell differentiation was induced with a mixture (DMI) of 0.5 mM 3-isobutyl-1-methylxanthine, 100 µM indomethacin, 0.25 µM dexamethasone and 167 nM insulin in DMEM containing 10% FBS. 3-isobutyl-1-methylxanthine (MIX), dexamethasone (DEX), indomethacin, and Oil-red O were obtained from Sigma-Aldrich (St. Louis, MO, USA). This medium was changed every 2 days. BPEs were treated into the culture medium containing adipocytes at day 0. Cells were treated with 0, 50, 200, or 300 µg/ml of BP extracts. After treatment with BP for 4 or 7 days, the 3T3-L1 adipocytes were lysed for Western blotting analysis. To analyze cell viability, cytotoxicity of the BPE was evaluated by 3-(4, 5-demethylthiazol-2-yl)-2, 5-diphenyltetrazolium bromide (MTT) assay.

### Oil-red O Staining and Triglyceride Assay

For Oil-red O staining, cells were washed gently with phosphate-buffered saline (PBS) and stained with filtered Oil-red O solution (60% isopropanol and 40% water) for 30 min. After staining, the Oil-red O staining solution was removed, and the plates were rinsed with water and dried. The stained lipid droplets were viewed on an Olympus microscope (Tokyo, Japan). To analyze the content of cellular triglycerides, the cells were washed with PBS, scraped into 200 µl PBS and sonicated for 1 min. When the elective PI3K inhibitor, 10 µM LY294002 (Sigma-Aldrich, St. Louis, MO, USA) was used, 3T3-L1 cells were incubated with or without LY294002 in the presence or absence of the BPE for 6 days. The lysates were assayed for their total triglyceride content using assay kits from Sigma-Aldrich (St Louis, MO, USA) and for cellular protein using the Bio-Rad protein assay (CA, USA). The results were expressed as mg of triglyceride per mg of cellular protein.

### RT-PCR

Total RNA was isolated from 3T3-L1 adipocytes using Trizol reagent (Invitrogen, CA, USA). One microgram of total RNA was subjected to first strand cDNA synthesis with oligo (deoxythymidine) primers and Superscript II reverse transcriptase (Invitrogen, CA, USA). The target cDNA was amplified using the following sense and antisense primers: sense 5′-GACTACGCAACACACGTGTAACT-3′ and antisense 5′-CAAAACCAAAAACATCAACAACCC-3′ for C/EBPβ; sense 5′-TTTTCAAGGGTGCCAGTTTC-3′ and antisense 5′-AATCCTTGGCCCTCTGAGAT-3′ for PPARγ; sense 5′-TTACAACAGGCCAGGTTTCC-3′ and antisense 5′-GGCTGGCGACATACAGATCA-3′ for C/EBPα; Control detection of β-actin was performed with sense (5′-GACAACGGCTCCGGCATGTGCAAAG-3′) and antisense (5′-TTCACGGTTGGCCTTAGGGTTCAG-3′) primers. The amplification cycles were 95°C for 50 sec, 55°C for 1 min and 72°C for 50 sec. After 30 cycles, PCR products were separated by electrophoresis on 1.5% agarose gel for 30 min at 100 V. Gels were stained with 1 mg/ml ethidium bromide visualized by UV light using BIO-RAD Gel Doc image analysis software (BIO-RAD Laboratories Inc., CA, USA).

### Western Blot Analysis

Western blotting was performed according to standard procedures. Briefly, cells were lysed in lysis buffer containing 50 mM Tris-HCl (pH 8.0), 0.4% Nonidet P-40, 120 mM NaCl, 1.5 mM MgCl_2_, 0.1% SDS, 2 mM phenylmethylsulfonyl fluoride, 80 µg/ml leupeptin, 3 mM NaF and 1 mM DTT. Cell lysates were separated by 10% SDS-polyacrylamide gel electrophoresis, transferred onto a polyvinylidene fluoride membrane (Amersham Pharmacia, England, UK), blocked with 5% skim milk and hybridized with primary antibodies. The PPARγ, C/EBPβ, C/EBPα, aP2, FAS, Akt, and GSK3β antibodies were purchased from Cell Signaling, and the monoclonal β-actin antibody was purchased from Chemicon. The HRP-labeled mouse anti-rabbit IgG antibody was purchased from Jackson ImmunoResearch. The chemiluminescence kit was purchased from Pierce (Rockford, IL). After incubation with horseradish-peroxidase-conjugated secondary antibody at room temperature, immunoreactive proteins were detected using a chemiluminescent ECL assay kit (Amersham Pharmacia, UK) according to the manufacturer’s instructions.

### Animal Experiments

The study protocol was approved by the Animal Care and Use committee of Gyeongsang National University (Approval Number: GNU-120820-R0035). Five-week-old male Sprague-Dawley (SD) rats weighing approximately 150 g were purchased from the Central Lab. Animal Inc. (Seoul, Korea). The rats were acclimatized to the experimental facility for 1 week. The rats were divided into 4 groups of 10 and individually housed in polycarbonate cages in a room maintained at 22°C and 55% relative humidity. The room was exposed to alternating 12 h periods of light and dark. All of the rats were allowed free access to food and water for 5 weeks. Food intake was measured daily, and the rats were weighed every two days. Obese rats were generated by feeding rats a high-fat diet (HFD). Group A (ND) was maintained on a normal diet (ND) based on a commercial diet (#55VXT0038, Samyang Co, Korea); Group B (HFD-SBPE) was fed an HFD, and BPE (60 mg/kg BW) was administered through the gastrointestinal tract; Group C (HFD-LBPE) was fed an HFD, and BPE (150 mg/kg BW) was administered through the gastrointestinal tract; and Group D (HFD) was fed an HFD based on a commercial diet (rodent diet with 60% kcal fat, Research Diet, Korea).

### Biochemical Assays

After 5 weeks on experimental diets, the rats were euthanized, and the tissues were dissected out and analyzed. The body and fatty tissue weights were measured with sensitivity limits of 0.1 g and 0.01 g, respectively. The body mass index was calculated by dividing the weight (g) by the square of body length (cm^2^). Blood was collected from each rat, stored at 37°C for 30 min, and centrifuged at 4000×g at 4°C for 10 min to obtain plasma. The epididymal fat pad and perirenal fat pad were excised, weighed and stored at −20°C until assayed. The concentrations of plasma triglyceride (TG), total cholesterol (TC), and high-density lipoprotein (HDL)-cholesterol were assayed enzymatically using commercial kits (Asan phams, Co., Korea).

### Statistical Analysis

The data are expressed as the means ± SD. The significant differences in the treatment means were determined using ANOVA and Duncan’s multiple range test at p<0.05.

## Results

### Total Phenol Content (TPC) and Total Flavonoid Content (TFC) of BPE

The TPC and TFC contents of BPE were found to be (131.3±14.47) of quercetin equivalent/g, and (113.4±0.72) of quercetin equivalent/g extract, respectively (Table 1).

**Table 1:** Antioxidant capacities, total phenolic and flavonoids content of BP extracts.

	DPPH^a^	HRSA^b^	SRSA^c^	TPC^d^(mgQE/g)	Flavonoid^e^(mgQE/g)
Blueberry Peel	19.4±2.71^y^	8.2±2.05^z^	4.6±1.03^z^	131.3±14.47^y^	113.4±0.72^x^
Positive control1)	80.5±0.28^x^	34.6±3.25^x^	31.1±3.20^x^	–	–

a DPPH, DPPH radical scavenging activity;

b HRSA, hydroxyl radial scavenging activity;

c SRSA, superoxide anion radical scavenging activity;

d TPC, total phenolic acid. Total phenolic acid and total flavonoid content expressed as milligrams of quercetin equivalent (QE)/g of extract.

1) The positive controls of DPPH, HRSR and SRSA were ascorbic acid, ascorbic acid and quercetin, respectively.

x–z The values are presented as the means ± SD. P<0.01 represents a significant difference between the samples (n = 4).

### DPPH, Hydroxyl, and Superoxide Anion Radical Scavenging Activity

DPPH radical scavenging activity of BPE was shown in table 1. The extract exhibited potent DPPH radical scavenging activity, but comparatively less than the ascorbic acid. Superoxide radical scavenging activity of BPE was assessed by the reduction of nitroblue tetrazolium. The BP extract inhibited the superoxide radical generation. In hydroxyl radical scavenging activity, the extract was found to possess antioxidant activity but less potent when compared to ascorbic acid. The scavenging activities of superoxide anion radicals and hydroxyl radicals are shown in Table 1.

### Inhibitory Effect of BPE on Adipogenesis

**Figure 1:**
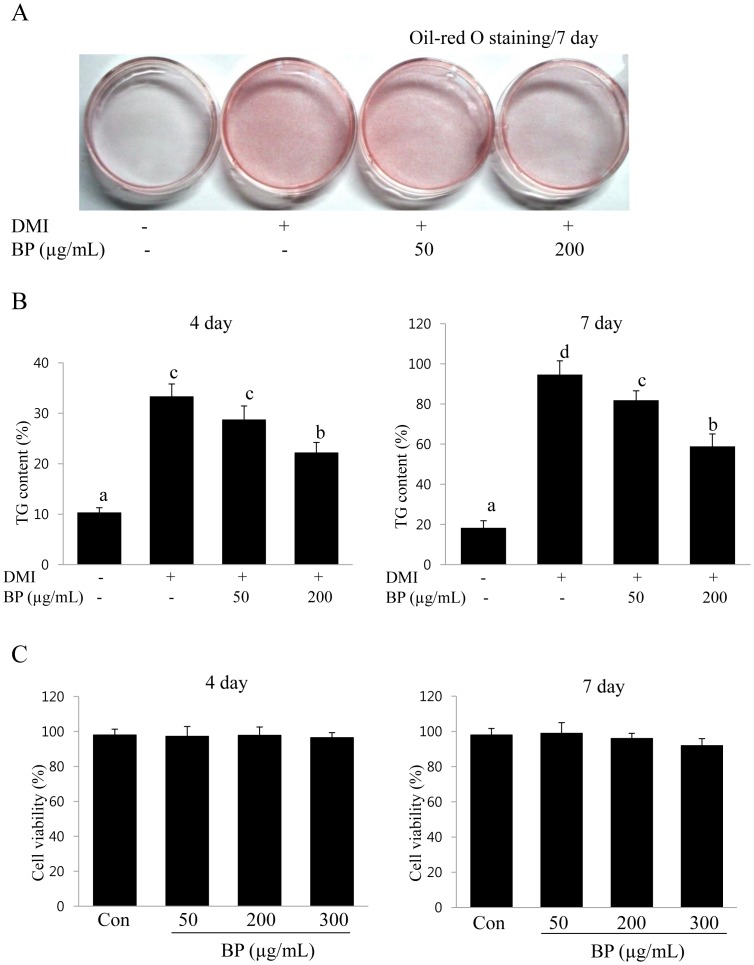
BPE inhibits intracellular lipid accumulation in 3T3-L1 cells. (A) Hormone-induced differentiation of 3T3-L1 adipocytes was repressed by BPE. Confluent 3T3-L1 preadipocytes differentiated into adipocytes in medium containing different concentrations of BPE for 7 days (from day 0 to 7). Oil-red O staining was performed on day 7. DMI: fully differentiated-adipocytes (0.5 mM 3 IBMX, 100 µM indomethacin, 0.25 µM dexamethasone and 167 nM insulin). BPE: blueberry peel extracts. (B) BPE reduced TG accumulation in differentiated 3T3-L1 cells. The data shown are representative of at least three independent experiments. The values are presented as the means ± SD. Bars with different letters are significantly different (p<0.05) as determined by Duncan's multiple range test. (C) The effect of BP on cell viability in preadipocytes. 3T3-L1 preadipocytes were incubated with BP extracts (0–300 µg/mL) for 7 days. Cell viability after treatment with BP was determined by the MTT assay. The values are presented as the means ± S.D. The data shown are representative of at least three independent experiments.

To examine the anti-adipogenic effects of BPE, 3T3-L1 preadipocytes were treated with BPE for 7 days. The anti-adipogenic effect of BPE on the induction of differentiation markers in 3T3-L1 cells was measured at the middle (day 4) or the end (day 7) of the differentiation experiment. The differentiation of preadipocytes into adipocytes is associated with an increased number of Oil-red O stained cells due to lipid accumulation. Microscopic observations of the Oil-red O staining revealed a gradual reduction in the number of lipid droplets as the concentration of BPE increased (Fig. 1A). The amount of accumulated triglycerides was analyzed on day 4 or 7, and the cells treated with 200 µg/ml BPE had a significantly lower lipid content on day 7 (Fig. 1B). The inhibitory effects of BP on triglyceride accumulation during adipogenesis were dose-dependent and treating differentiated cells with BPE (200 µg/ml) decreased triglyceride levels by 37.7% in 7 days (Fig. 1B). This anti-adipogenic effect was achieved at concentration that did not affect cell viability according to the MTT assay (Fig. 1C). These results indicate that BPE effectively blocks adipocyte differentiation in 3T3-L1 preadipocytes.

### Inhibitory Effect of BP on the Expression of Adipogenic-specific Genes and Protein

**Figure 2:**
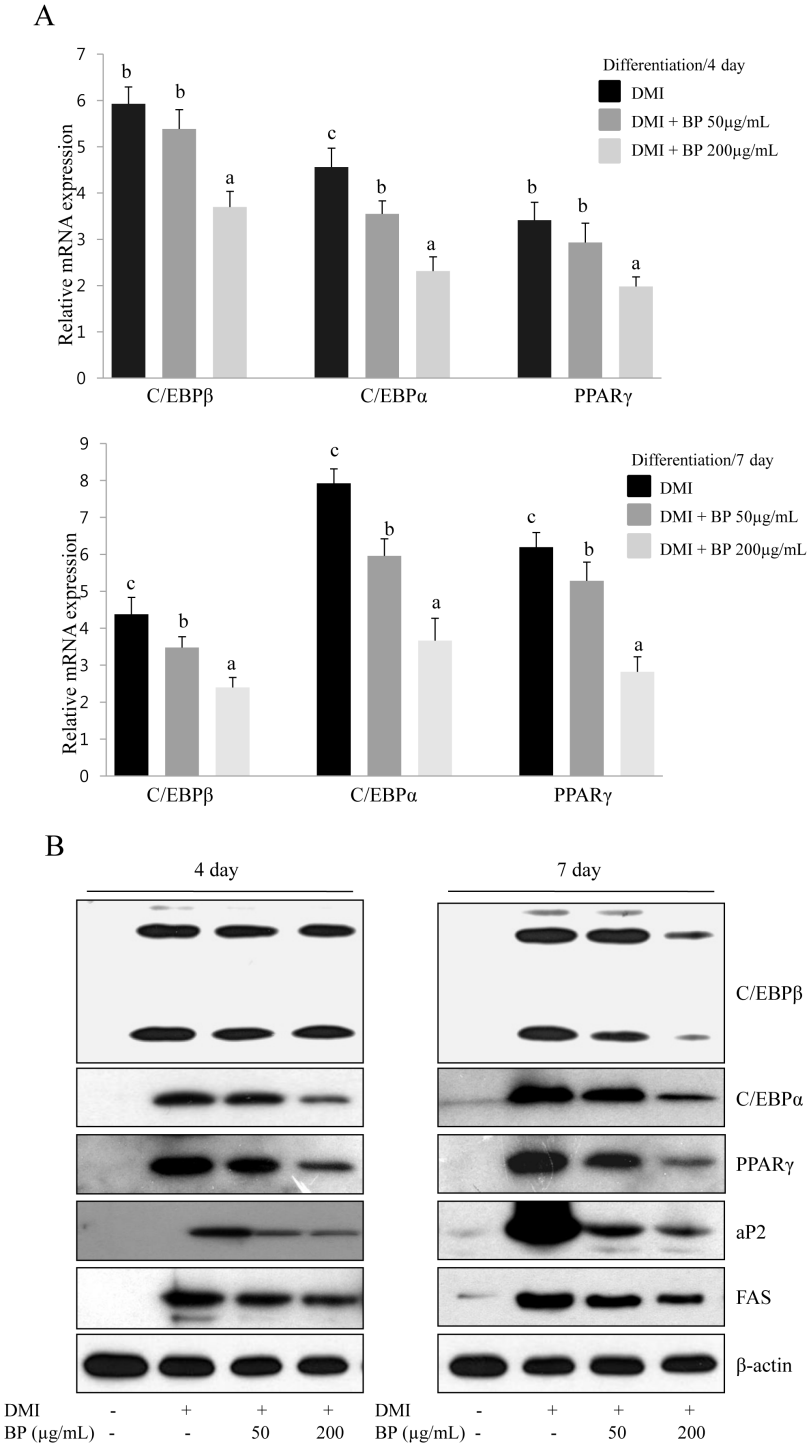
Effect of BP on the expression of adipogenic genes in 3T3-L1 adipocytes. 3T3-L1 preadipocytes were differentiated into adipocytes in DMI medium in the absence or presence of 50 µg/mL or 200 µg/mL BPE for 4 or 7 days. (A) BPE inhibited the expression of adipocyte-specific transcription factors during differentiation. The gene expression analysis was performed by RT-PCR, and all of the gene transcripts were normalized using β-actin as a control. All of the experiments were performed in three independent experiments. Bars with different letters are significantly different (p<0.05) as determined by Duncan's multiple range test. (B) BP reduced the expression of adipogenesis-related genes in 3T3-L1 adipocytes. Total cell lysates were isolated from 3T3-L1 adipocytes at day 4 or day 7 after induction of differentiation. Western blotting analysis was performed as described in the Materials and Methods.

To investigate the effects of BP extracts on the differentiation of 3T3-L1 preadipocytes, 3T3-L1 cells were differentiated in DMI medium containing BPE at 50 µg/mL or 200 µg/ml for 7 days. The effect of BPE on the expression of C/EBPα, C/EBPβ, and PPARγ was examined using RT-PCR and Western blotting. We observed that the mRNA levels of C/EBPβ, C/EBPα, and PPARγ were reduced by treating differentiated 3T3-L1 with BP extracts and the inhibitory effects of BP exhibited a dose-dependent pattern (Fig. 2A). Furthermore, our Western blotting analysis also showed that the expression of C/EBPβ, C/EBPα, and PPARγ proteins decreased in response to BP extracts and this effect was strongly suppressed 7 days after the initiation of BPE treatment (Fig. 2B). We further investigated whether BP could regulate the protein expression of adipogenic target genes such as aP2 and FAS. The addition of BP extracts during adipocyte differentiation reduced the expression of aP2 and FAS in a dose-dependent manner compared with control adipocytes that were not treated with BPE extracts (Fig. 2B). These results suggest that BPE extracts significantly induce the down-regulation of adipogenic transcription factors, which play a critical role in adipocyte differentiation.

### The Effect of BP on the Regulation of Akt and GSK3β during Adipocyte Differentiation

**Figure 3:**
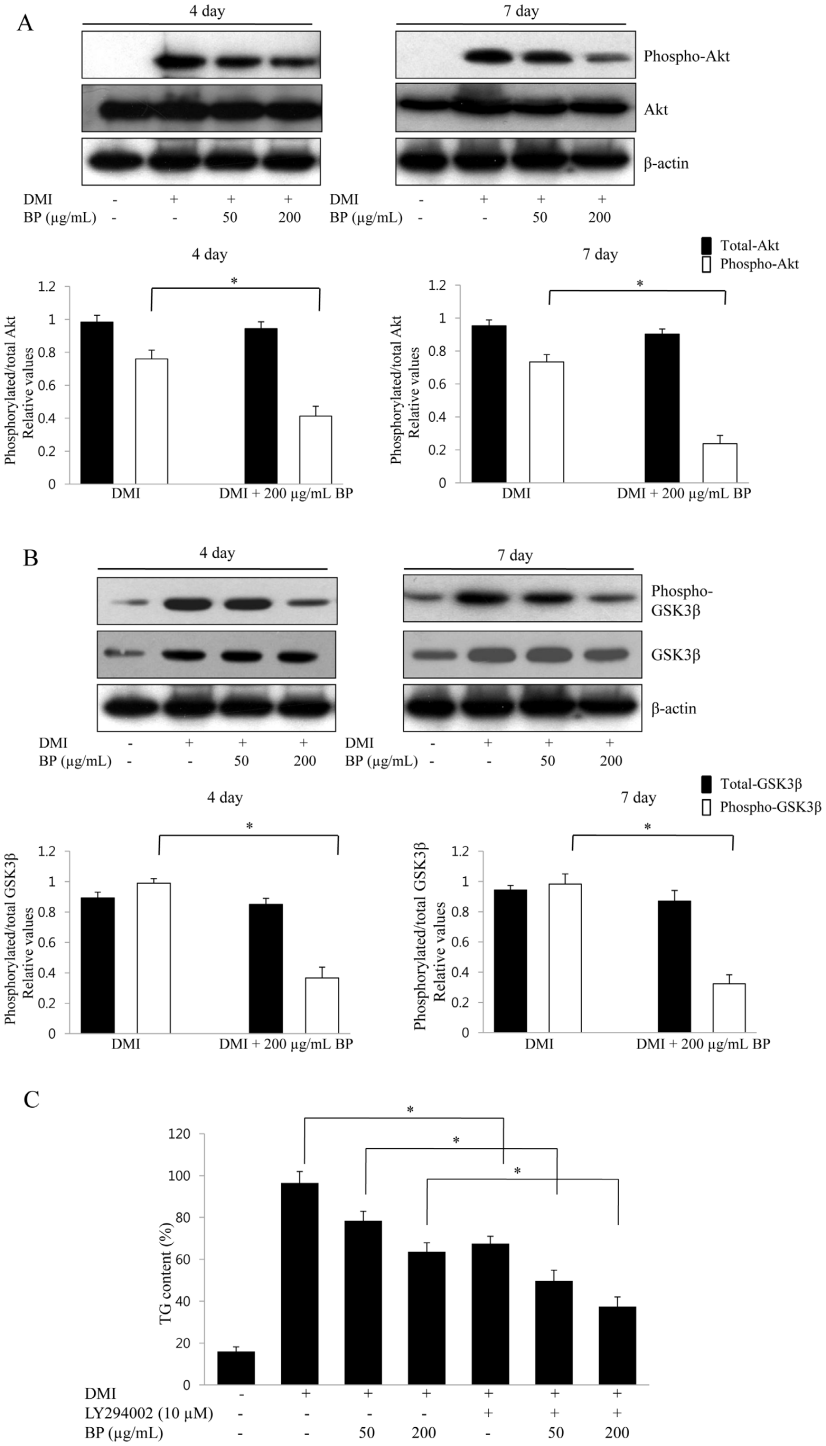
Effect of BP on phosphorylation of Akt and GSK3β in 3T3-L1 adipocytes. (A) Effect of BP on Akt activation in 3T3-L1 adipocytes. 3T3-L1 adipocytes were treated with BP extracts at the indicated concentrations and the phosphorylation levels for Akt was determined by Western blotting analysis. The data are presented as the means ± SD values for at least three independent experiments. *P<0.05. (B) Effect of BP on GSK3β activation in 3T3-L1 adipocytes. 3T3-L1 adipocytes were treated with BP extracts at the indicated concentrations, and the phosphorylation levels for GSK3β were determined by Western blotting analysis. The data are presented as the means ± SD values of at least three independent experiments. *P<0.05. (C) Effects of the PI3K/Akt inhibitor LY294002 (10 µM) on BP-induced inhibition of adipocyte differentiation in 3T3-L1 cells. 3T3-L1 cells were treated with BPE during differentiation in the presence or absence of the LY294002. The intracellular lipid accumulation was measured by triglyceride assay. Data are expressed as mean ± SD of three independent experiments. *P<0.05.

Akt is important in glucose regulation and lipid metabolism in insulin signaling, and GSK3β is a downstream target of Akt in adipocyte differentiation. To study the molecular mechanisms underlying the BPE-induced anti-adipogenic effect, we examined the effects of BP extracts on the levels of phosphorylated Akt during adipocyte differentiation of 3T3-L1 cells. We analyzed 3T3-L1 cell lysates treated with BP extracts at various concentrations (0, 20, 200 µg/mL). The phosphorylation of insulin-stimulated Akt was reduced after treatment with BP extracts, although the expression levels of wild type Akt did not change compared with the controls (Fig. 3A). The addition of BP extracts decreased the level of phospho-Akt in a dose-dependent manner compared with the differentiated control cells, and 200 µg/ml of BP extracts significantly inhibited the expression of phospho-Akt (Fig. 3A). In addition, the amount of insulin-stimulated phosphorylated GSK3β decreased with the addition of BP extracts while wild type GSK3β was not affected by the BP extracts compared with insulin only (Fig. 3B). These results demonstrate that BPE treatment inhibits the phosphorylation of Akt, which suppresses the phosphorylation of its substrate kinase GSK3β. To further investigate whether PI3K/Akt signalling pathway is involved in the inhibition of adipocyte differentiation by BP, 3T3-L1 cells were treated with BP in adipogenic medium in the presence or absence of LY294002 (10 µM), a specific inhibitor of PI3K/Akt for 6 days. Differentiated 3T3-L1 cells after treatment with an MDI mixture for 6 days had a much higher level of lipid droplets than nondifferentiated cells, as shown by the increase in intracellular triglyceride content (Fig. 3C). BPE reduced cellular lipid accumulation in a dose-dependent manner in 3T3-L1 adipocytes. As expected, incubation of 3T3-L1 adipocytes with LY294002 markedly inhibited the DMI-induced adipocyte differentiation of 3T3-L1 cells. Moreover, triglyceride contents were significantly decreased in LY294002 plus BPE-treated cells compared to that of LY294002 alone-treated cells (Fig. 3C). Triglyceride accumulation was strongly inhibited in the presence of BP, suggesting that BPE prevent adipocyte differentiation through an inhibition effect of PI3K/Akt signalling pathway in 3T3-L1 cells.

### Changes in Body Weight and Body Fat in HFD-induced Obese Rats

**Figure 4:**
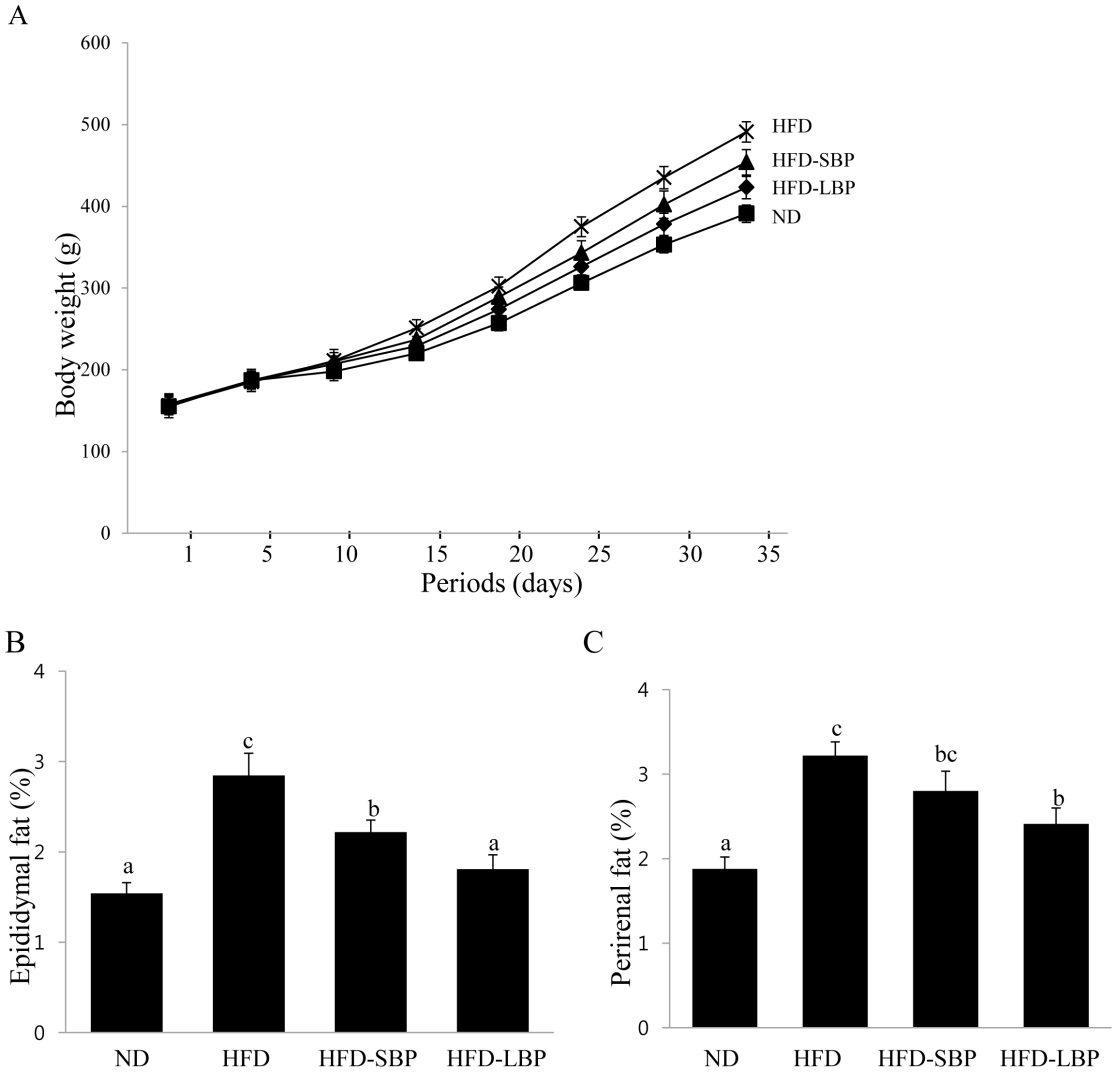
Effects of BP extracts on body weight in HFD-induced obese rats. (A) ND groups (▪) were fed normal diet (ND), HFD-SBP groups were fed HFD plus BPE (60 mg/kg BW, ▴), HFD-LBP groups (♦) were fed HFD plus BPE (150 mg/kg BW), and HFD groups (×) were fed high-fat diet. The body weight was measured twice a week. Body weights at the end of the experiments were significantly different between the HFD and ND (P<0.01) and HFD-BP groups (P<0.05). (B, C) BPE treatment decreased perirenal and epididymal fat weights in HFD-induced obese rats. The weights of the perirenal and epididymal fatty tissue were calculated by dividing the fatty tissue weight by body weight (fatty tissue/body weight x 100). The values are expressed as the means ± SD. Bars with different letters are significantly different (p<0.05) as determined by Duncan's multiple range test.

BP extracts inhibited adipocyte differentiation in 3T3-L1 preadipocytes, suggesting that BPE might suppress HFD-induce obesity. To examine whether BPE has an anti-obesity effect in rats on a HFD, we supplemented the high fat diet with BP extracts. The HFD-induced obese rats were weighed after BP extracts were administered through the gastrointestinal tract at a concentration of 60 mg/kg BW/day (HFD-SBP) or 150 mg/kg BW/day (HFD-LBP) for 5 weeks. After 5 weeks, all of the rats on a high fat-diet were 25.5% heavier compared with normal-diet controls (ND) (Fig. 4A). In contrast, rats on a high-fat diet supplemented with BP were 8.3% (HFD-SBP) and 15.8% (HDF-LBP) lighter than rats fed only a high-fat diet. Although there was no significant difference in food intake among the groups during the experimental diet period, the body weight gain of the HFD-LBP group was significantly lower than the weights of the HFD groups (Fig. 4A). The fatty tissue mass in epididymal and perirenal adipose tissue was also significantly lower in the LBP-fed rats compared to the high-fat diet rats (Fig. 4B, C). The addition of BPE did not induce liver toxicity in the HFD-induced obese rats (data not shown). Thus, BPE effectively inhibits high-fat diet-induced body weight gain and adipose tissue mass in rat.

### Effect of BPE on the Serum Lipid Profile of HFD-induced Obese Rats

**Figure 5:**
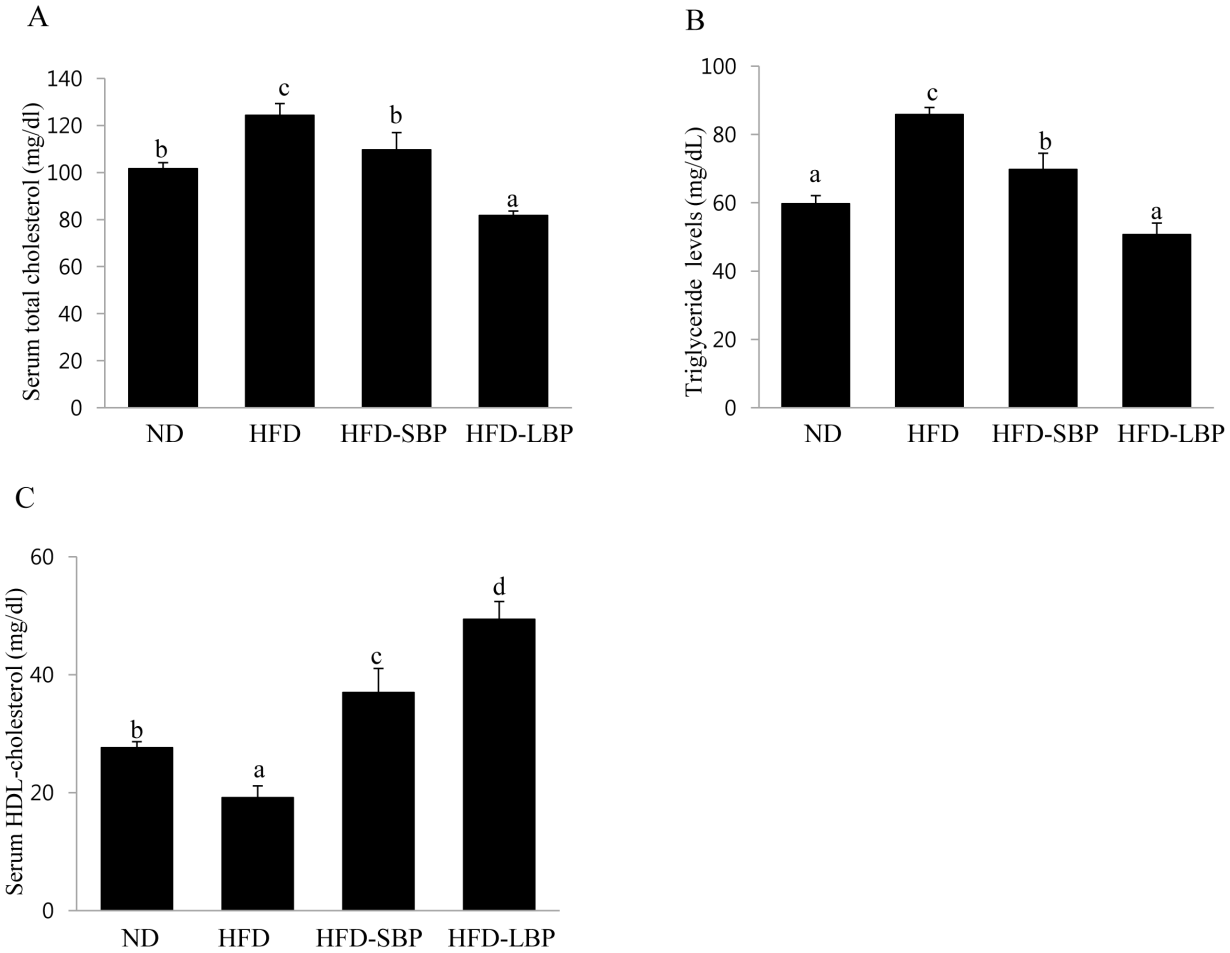
Effect of BP on lipid contents in the HFD-induced obese rats. (A, B, C) Significant decreases in the levels of serum triglyceride and total cholesterol were observed in the BPE-treated groups compared with HFD-induced obese rats. HDL-cholesterol levels in the BP groups were increased compared with the HFD groups. The values are expressed as the means ± SD. Bars with different letters are significantly different (p<0.05) as determined by Duncan's multiple range test.

Serum total cholesterol levels in rats fed BPE were reduced by 11.5% (SBP) and 31.5% (LBP) compared with those in HFD-fed rats (Fig. 5A). The addition of BB in the SBPE or LBPE groups decreased triglyceride levels by 20% and 36%, respectively, compared with the levels found in rats fed a HFD (Fig. 5B). After 5 weeks of a high-fat diet, the serum HDL-cholesterol levels decreased compared with the normal diet control group. However, the serum HDL-cholesterol levels in the LBPE group increased by approximately 155% compared with the levels from rats on a HFD (Fig. 5C).

## Discussion

In the present study, we evaluated the effects of BP on adipocyte differentiation as well as its inhibitory mechanisms on adipogenesis in 3T3-L1 cells and anti-obesity activities in HFD-induced obese rats. Our results demonstrated that BP exhibited potent antioxidant activity, total phenolic and flavonoid contents. BP exerted antiadipogenic effects through inhibition of C/EBPβ, C/EBPα, and PPARγ expression and the Akt signaling pathway in 3T3-L1 cells, leading to decreased body weight and fat tissue mass in HFD-induced obese rats.

Adipocyte differentiation and fat accumulation are associated with the occurrence and development of obesity [26]. Hyperplastic obesity is caused by an increase in the number of fat cells relative to the increase in adipose tissue mass. A reduction of adiposity is related to the inhibition of angiogenesis along with a reduction of adipocyte numbers and the lipid content of adipocytes. The differentiation of preadipocytes into adipocytes is regulated by a complex network of transcription factors. In the present study, BPE treatment strongly suppressed C/EBPβ mRNA and protein expression and markedly reduced the expression levels of C/EBPα and PPARγ compared with those in differentiated control cells. Furthermore, treatment with BB extracts attenuated lipid accumulation as determined by Oil-red O staining and a triglyceride accumulation assay. C/EBPβ was induced immediately after differentiation, and C/EBPα and PPARγ are master regulators of adipogenesis; their maintenance is critical to the progression of the final stages of adipocyte differentiation [6], [27]. Thus, these results indicated that BB extracts significantly reduced lipid accumulation by down-regulating the adipogenic transcription factors that play a critical role in adipocyte differentiation. Moreover, PPARγ is activated by fatty acid, and fat accumulation is associated with PPARγ activation [28]. PPARγ and C/EBPα activate the expression of genes involved in adipogenesis, such as aP2, FAS, and LPL, to trigger the synthesis of fatty acids and triglycerides [5], [7]. During the terminal phase of differentiation, adipocytes dramatically increase lipogenesis and become sensitive to insulin. The activation of genes involved in TG metabolism, including ACC, FAS and aP2, are increased 10–100 fold [29], [30]. In these studies, the expression of aP2 and FAS was significantly lower in 3T3-L1 cells treated with BP extracts compared with terminally differentiated 3T3-L1 adipocyte control cells. Taken together, the reductions of aP2 or FAS expression are due to the down-regulation of C/EBP and PPAR family members, which not only slow down the de novo synthesis of fatty acids and TG but also inhibit the differentiation of early differentiating preadipocytes and lipogenesis in mature adipocytes.

The serine/threonine kinase Akt is particularly important in mediating adipocyte differentiation and the metabolic actions of insulin. Akt phosphorylates and regulates a large number of substrates involved in a diverse array of biological processes [31], many of which could contribute to the role of Akt in adipocyte differentiation. GSK3β is a critical downstream signaling protein for the phosphoinositide 3-kinase (PI3K)/Akt pathway. To elucidate the molecular mechanism underlying the BPE-induced anti-adipogenesis of 3T3-L1 preadipocytes, we measured the protein levels of phosphorylated Akt and its substrate kinase, GSK3β. Our observations showed that the serine phosphorylation of Akt was decreased by BB extracts in a dose-dependent manner and subsequently attenuated the levels of phosphorylated GSK3β. These data indicated that inhibiting Akt phosphorylation reduced the phosphorylation of downstream signaling components. Thus, our results strongly demonstrated that insulin-mediated Akt phosphorylation and activation was inhibited by BP extract treatment, which mainly affected the reduced accumulation of triglyceride formation by inhibiting the PI3K/Akt pathway during the differentiation of 3T3-L1 preadipocytes into adipocytes.

Interestingly, GSK3β is a critically important protein kinase in adipocyte differentiation because it phosphorylates either C/EBPβ or C/EBPα. The inhibition of GSK3β phosphorylation (serine 9) leads to C/EBPβ phosphorylation and inactivation [32], which is consistent with the negative regulation of C/EBPβ by GSK3β phosphorylation. Shim et al. showed that the GSK3β-mediated phosphorylation of C/EBPα targets it for proteasomal degradation [33], and another study also demonstrated that the Fbxw7-dependent degradation of C/EBPα was dependent on the phosphorylation of Thr222 and Thr226, which are GSK3β phosphorylation sites [34]. In addition, other studies have demonstrated that the phosphorylation of serine 9 in GSK3β increases following insulin treatment, and its activity is repressed by insulin and lithium chloride (LC) [35]. The treatment of 3T3-L1 cells with LC in the differentiation medium inhibited PPARγ expression and adipocyte differentiation [35]. The forced expression of PPARγ in Akt-deficient mouse embryonic fibroblasts rescued their severe adipogenesis defect [10], which supports the essential role of PPARγ induction downstream of Akt. Therefore, our results indicate that the inhibition of Akt phosphorylation and activation by BB block insulin-induced adipocyte differentiation in 3T3-L1 preadipocytes. Moreover, co-treatment of PI3K/Akt inhibitor, LY294002 and BPE exhibited more significant inhibitory effect on triglyceride accumulation in 3T3-L1 cells when compare with the LY2904002 alone treatment cells. These results strongly indicated that BP suppressed the adipogenic induction of lipid accumulation through an inhibition of PI3K/Akt-dependent signalling pathway. Taken together, this observation implies that there is an important association between PI3K/Akt/GSK3β mediated-signaling and the C/EBPβ, C/EBPα, and PPARγ transcription factors in the induction of 3T3-L1 adipocyte differentiation. Therefore, these results suggest that BB may inhibit Akt, which leads to suppressed adipogenesis through the inhibition signaling cascades, including C/EBPβ, C/EBPα, PPARγ, during 3T3-L1 adipocyte differentiation.

BB has been known as a treatment for obesity and diabetes-related complications [36]. The blueberry fruit is rich in phenolic compounds such as hydrocinnamic acids, flavonoids, and proanthocyanidins [37]. In the present study, the results revealed that BPE had effective capacity of scavenging for DPPH, superoxide anion, and hydroxyl radicals and correlated with potent phenol and flavonoid contents, thus suggesting its antioxidant potential. Blueberry phenols have various physiological functions that include antioxidant, anti-cancer, and anti-diabetes roles [38]. Blueberry pomace was effective in ameliorating fructose-induced metabolic abnormalities [19]. Rodriguez-Mateos et al. showed that BB supplementation improves vascular reactivity and lowers blood pressure in high fat fed rats [39]. Biotransformed BB juice increased AMP-activated protein kinase phosphorylation and glucose uptake in muscle cells, but inhibited adipogenesis [40]. BP extracts protect against adipose tissue inflammation and insulin resistance, which provide metabolic benefits to combat the obesity-associated pathology [41].

In this study, we used an HFD-induced obesity rat model to investigate the anti-obesity effects of BP extracts. All of the rats were maintained on normal diet (ND) for 1 week and then fed ND, HFD, HFD plus BB (60 mg/kg BW, SBPE), or HFD plus BPE (150 mg/kg BW, LBPE) for 5 weeks. The weekly food intake was similar between the groups. The body weights of HFD-induced obese rats were monitored after daily oral administration of BPE for 5 weeks. Our results showed that body weights of rats fed an HFD plus SBPE were slightly reduced and were comparable to those of rats fed an ND when measured at the end of the study. However, the body weights of rats on an HFD plus LBPE were significantly lower. Consistent with the changes in body weights, BPE feeding obviously decreased the weight of epididymal or perirenal adipose tissues in HFD-induced rats. These results strongly indicated that the BPE-mediated decrease in body weight was due to a reduction in adipose tissue weight, independent of food intake. Similarly, BPE supplements also caused a decrease in the triglyceride concentrations and total cholesterol in the blood of HFD-induced obese rats. We observed that rats fed an HFD supplemented with BPE for 5 weeks significantly increased the levels of HDL-cholesterol compared with rats fed an HFD alone, indicating that BPE efficiently regulates triglyceride and cholesterol metabolism in HFD-induced obese rats. These results partially agreed with previous reports in hamsters [42], pigs [43], and rats [44]. The total cholesterol, a combination of low- and high-density lipoprotein (LDL and HDL, respectively) cholesterol circulating in the blood, is one of the most commonly examined measurements in a lipid profile. HDL cholesterol is considered to be good cholesterol, and high levels of HDL are a good indicator of a healthy heart because less cholesterol is available to attach to blood vessels. Thus, the addition of a BPE supplement is critical in reducing the cholesterol levels in HFD-induced obese rats.

In general, a high-fat diet is associated with obesity-mediated insulin resistance [45] and with elevated total serum cholesterol and triglyceride levels and inhibiting the absorption of triglycerides have an important role in weight loss [46]. In this study, BP diet tended to decrease the serum level of total cholesterol and triglyceride in comparison with rat given the high-fat control fat. The present results demonstrated that BP plays an important role in adipocyte lipid metabolism and their antiobesity actions. Therefore, we suggest that BP and its phenolic and flavonoid contents significantly exert antiadipogenic and obesity effects in cell and animal models.

In the present study, we examined the anti-obesity effects of BP on adipocyte differentiation and the associated mechanisms in 3T3-L1 cells. The addition of BP extracts reduced the expression of C/EBPβ and subsequently down-regulated the activation of the key transcriptional regulator C/EBPα and PPARγ in 3T3-L1 adipocytes. BP extracts treatment significantly attenuated lipid accumulation and adipocyte differentiation of 3T3-L1 cells in a dose-dependent manner. Moreover, BP extracts suppressed the activation of the adipogenic-specific genes such as aP2 or FAS compared with control adipocytes. Although we did not examine the effect of BP extracts on the inhibition of the Akt/GSK3β pathway in adipocytes from HFD-induced obese rats, our in vitro study supports our claim that the Akt/GSK3β pathway is involved. We demonstrated that BB extracts decreased lipid accumulation and adipogenic gene expression by inhibition of the PI3K/Akt/GSK3β pathway in 3T3-L1 preadipocytes that differentiated into adipocytes. Moreover, the administration of BP and its polyphenol extracts effectively prevented HFD-induced body weight gain and body fat accumulation, and decreased the plasma triglyceride and cholesterols levels in rats on a HFD. These results suggest that the anti-obesity effects of BP result from a decrease in adipogenesis and that BP has a beneficial effect by reducing the body weight gain in an obesity animal model.
